# Successful Photodynamic Therapy for a Case of Cutaneous Squamous Cell Carcinoma With Difficulty in Primary Healing After Surgery

**DOI:** 10.1002/ccr3.71674

**Published:** 2025-12-29

**Authors:** Jianhua Huang, Yongjun Cai, Hongwei Wang

**Affiliations:** ^1^ Department of Dermatology Huadong Hospital, Fudan University Shanghai China; ^2^ Department of Pathology Huadong Hospital, Fudan University Shanghai China

**Keywords:** 5‐aminolevulinic acid, cutaneous squamous cell carcinoma, photodynamic therapy, primary healing, surgical excision

## Abstract

Surgery combined with photodynamic therapy (PDT) for the treatment of large cutaneous tumors on the face has been proven to be effective, safe, and low recurrence. However, ulcerative cutaneous squamous cell carcinoma (cSCC) may not be able to achieve primary postoperative healing before subsequent photodynamics. Herein, we report that 5‐aminolevulinic acid photodynamic therapy (ALA‐PDT) perfectly solved the dilemma of non‐healing of left eyebrow cSCC after excision, providing inspiration and reference for clinical practice.

**Summary**

- Some skin tumors have high suture tension after operation, and such incisions can be left open without suture and solved by photodynamic therapy.

## 1 Introduction

Surgery combined with PDT is a safe, effective, and novel cosmetic therapy for skin cancer, which has been widely studied and applied due to its outstanding performance [1, 2]. However, sometimes with this combination therapy, the surgery can also be left open with no sutures, and the subsequent photodynamics can solve the problem perfectly.

## 2 Case History

**FIGURE 1:**
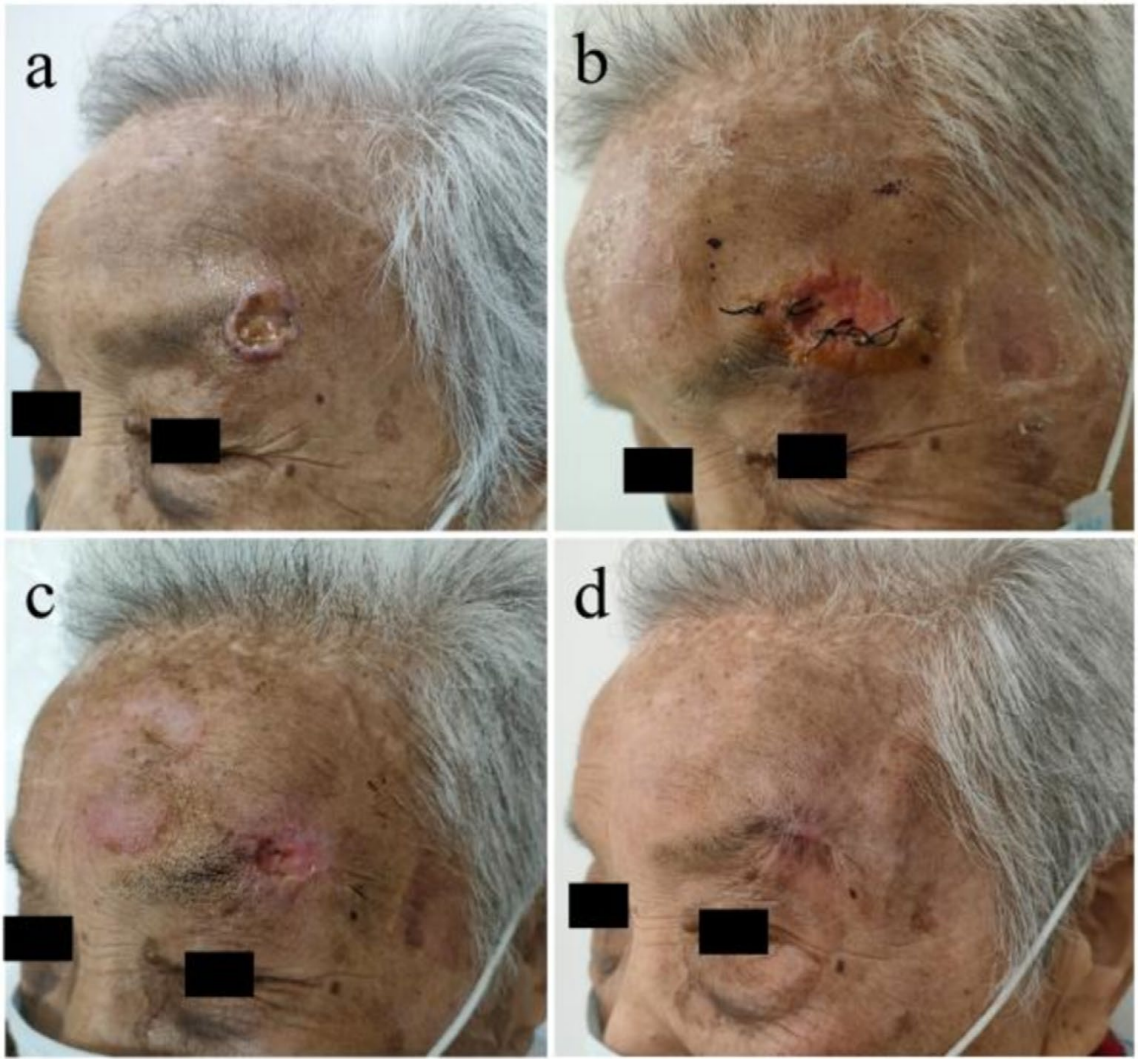
Left eyebrow cutaneous squamous cell carcinoma before and after treatment. (a) A crater‐like ulcer in the left eyebrow; (b) The state of spontaneous rupture after surgery; (c) A dramatic contraction of the incision after one session of ALA‐PDT; (d) Complete repair at the 2‐month follow‐up.

**FIGURE 2:**
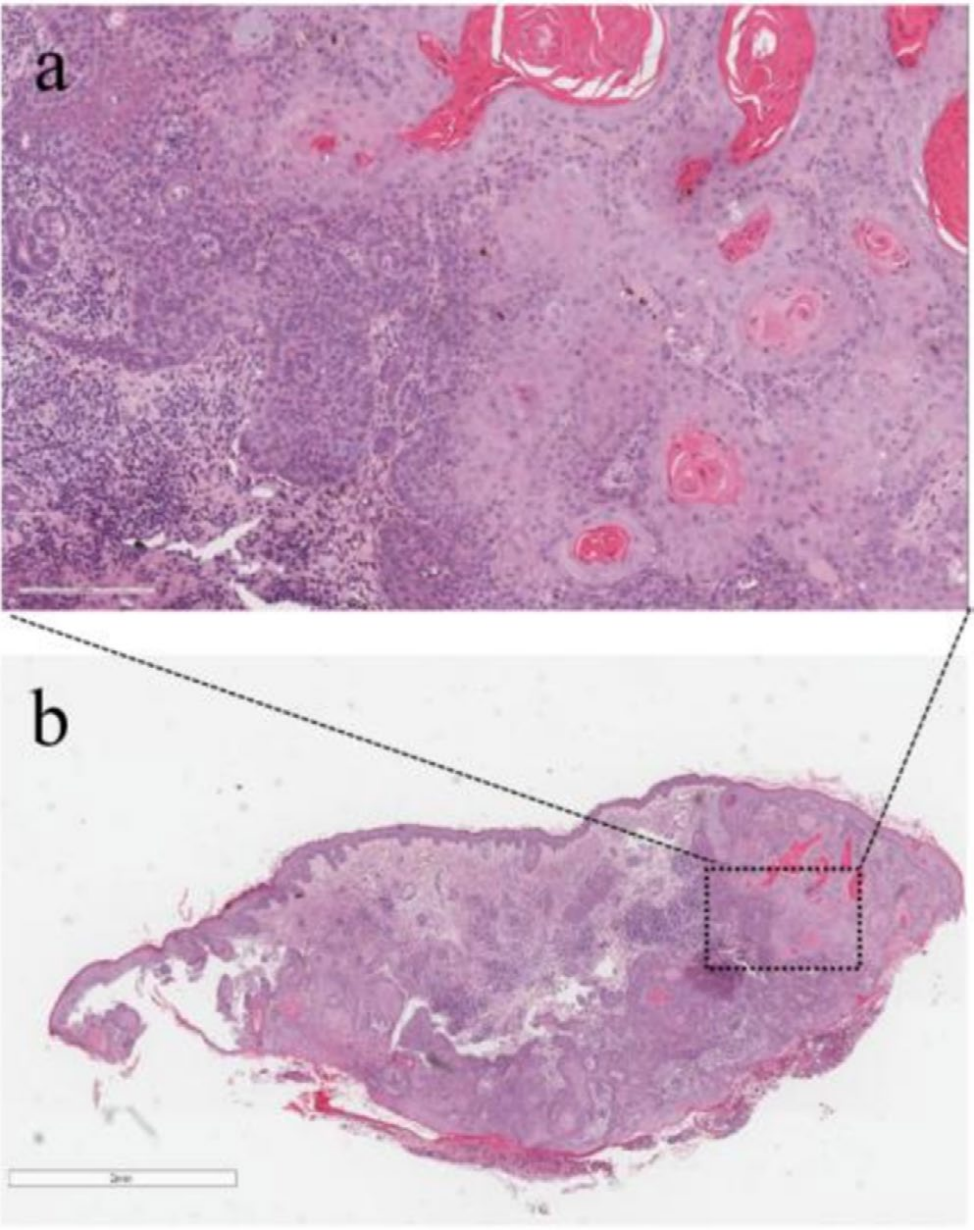
Pathological images. (a) Higher magnification displayed that the mesenchyma was infiltrated by heterotypic cells and inflammatory cells; (b) Low magnification revealed disordered cell arrangement and infiltration of squamous cell masses in the superficial and intermediate layers of the dermis. All sections were stained with hematoxylin and eosin.

An 85‐year‐old woman presented with a left eyebrow ulcer for more than 1 month. A physical examination revealed a deep circular ulcer about 1 cm in diameter on the left eyebrow, raised around the periphery and covered with pearl‐shaped nodules. The ulcer was pitted in the center, with an exudate of dirt and necrotic tissue at the base, accompanied by a foul odor (Figure 1a). Cervical and retro‐auricular examination revealed no palpable enlargement of lymph nodes. We learned that the patient was a farmer with a long history of outdoor farming and a weak awareness of sun protection. Based on the clinical features and medical history, cSCC was initially suspected. However, the possibility of a benign chronic ulcer, ulcerative basal cell carcinoma, tuberculous ulcer, and other diseases could not be ruled out. Further histopathologic study demonstrated obvious epidermal hyperplasia, disordered cell arrangement, and apparent cell abnormality. Atypical squamous cell clusters characterized by nuclear pleomorphism infiltrated the dermis, which was consistent with cSCC (Figure 2).

## 3 Treatment

Considering the large‐sized lesion and troublesome site for large‐scale surgery, we decided to resort to the strategy of surgical resection of the overall wound and subsequent PDT after first‐stage suture healing. After informed consent was obtained, the entire ulcer lesion was excised under local anesthesia. Pathological examination of the tumor tissue showed no malignant cells at the margin or bottom. On the second day after the operation, the incision was accidentally found to be split. After resuturing, the phenomenon of spontaneous incision dehiscence still occurred, and the expected first‐stage healing failed (Figure 1b). Faced with the dilemma that the incision could not heal at the first stage, we then resorted to PDT. 20% ALA cream composed of matrix cream and 5‐aminolevulinic acid (ALA, Shanghai Fudan Zhangjiang Biomedical Co. Ltd.) was evenly applied to the laceration. After 3 h, illumination was performed using a light‐emitting diode array (LED‐IB, China, 630 nm, 100 mW/cm^2^) for 25 min (approximately 150 J/cm^2^) at a distance of 10 cm from the surgical site. The frequency of ALA‐PDT was once a week for 3 times in total. Pain during the lighting process could be relieved by local anesthesia with a local injection of lidocaine.

## 4 Results

The wound became significantly smaller and shallower 1 week after the first ALA‐PDT (Figure 1c). A follow‐up of 2 months after the last session showed that complete recovery was observed with an unremarkable scar remaining (Figure 1d). Telephone follow‐up 1 year later revealed no recurrence.

## 5 Discussion

cSCC is the second most common non‐melanoma skin cancer, accounting for 20% of cutaneous carcinomas, with an estimated 1 million cases per year in the United States, a figure that continues to rise and is underestimated [3]. The incidence of cSCC is on the rise globally, with a 50%–300% increase in the Caucasian population over the past three decades, and the incidence in European countries is estimated to be twice its current level by 2030 [4]. Given its local aggressiveness and metastasis, cSCC apparently affects overall mortality [5]. It is responsible for the majority of skin cancer deaths in people over the age of 85 and is the second leading cause of skin malignancy death after melanoma [4].

Current treatments for cSCC contain surgery, cryotherapy, radiotherapy, chemotherapy, PDT, and anti‐PD1 immunotherapy (e.g., pembrolizumab, cemiplimab) [3]. The surgical procedure is the cornerstone of the management of cutaneous malignancies; however, it is traumatic and has limited effect on beauty. One of the clinical challenges is how to meet the needs of tumor therapies with a lower recurrence rate, less pain, and fewer side effects. PDT is a phototherapy in which a photosensitizer reacts with a specific wavelength of light to selectively destroy tumor lesions and has been considered one of the modalities for certain skin neoplasms. However, insufficient illumination penetration of PDT results in a lower clearance rate. Surgery supplemented by PDT combines the advantages of both approaches and compensates for their disadvantages. After complete resection of the tumor, PDT can reduce recurrence while maintaining the relative integrity of the local tissue and appearance as much as possible [6]. We believe that our previous report of a case in which surgery combined with ALA‐PDT successfully resolved a giant keratoacanthoma‐like SCC in an elderly woman could provide a reference for the management of this patient [7].

Both the advanced age of our case and her long history of sun exposure in the exposed area were risk factors for developing skin tumors such as cSCC [8]. The diagnosis of cSCC was made based on her medical history, typical lesions, and pathology. This case did not heal as expected after surgical resection. We speculated that the reason might be, on the one hand, the texture of the edge tissue of the incision was brittle, which could not be repaired normally, and on the other hand, the eyebrow arch tension was too large, which was not conducive to suture. For this postoperative state that could not achieve healing by first intention, PDT was the most appropriate choice because of its pro‐healing effect [9]. PDT itself can be used to treat tumors, and it also plays an important role in promoting repair. Our case healed completely after three sessions with no relapse during follow‐up, and no conspicuous scar mark was left.

## 6 Conclusion

This case indicates that the difficult‐to‐heal skin neoplasm postoperative wound is also an indication for PDT, which can be used confidently and boldly due to its potential of both anti‐tumor and wound healing.

## Author Contributions

**Jianhua Huang:** data curation, formal analysis, methodology, resources, writing – original draft, writing – review and editing. **Yongjun Cai:** resources. **Hongwei Wang:** supervision, validation, visualization.

## Funding

This paper was supported by the National Natural Science Foundation of China (No. 81572671), the Clinical Research Plan of SHDC (SHDC22022302), Shanghai Pujiang Program (22PJ1403400).

## Consent

Written informed consent was obtained from the patient for the publication of this case report and any accompanying images. A copy of the written consent is available for review by the journal's Editor‐in‐ Chief.

## Conflicts of Interest

The authors declare no conflicts of interest.

## Data Availability

The data that support the findings of this study are available on request from the corresponding author. The data are not publicly available due to privacy or ethical restrictions.
